# Anemia prevalence and incidence and red blood cell transfusion practices in aneurysmal subarachnoid hemorrhage: results of a multicenter cohort study

**DOI:** 10.1186/s13054-018-2089-7

**Published:** 2018-07-04

**Authors:** Shane W. English, Michaël Chassé, Alexis F. Turgeon, François Lauzier, Donald Griesdale, Allan Garland, Dean Fergusson, Ryan Zarychanski, Carl van Walraven, Kaitlyn Montroy, Jennifer Ziegler, Raphael Dupont-Chouinard, Raphaëlle Carignan, Andy Dhaliwal, Ranjeeta Mallick, John Sinclair, Amélie Boutin, Giuseppe Pagliarello, Alan Tinmouth, Lauralyn McIntyre, on behalf of the Canadian Critical Care Trials Group, V. McCredie, D. Scales

**Affiliations:** 1 Department of Medicine (Critical Care), The Ottawa Hospital, Civic Campus Room F202, 1053 Carling Avenue, Ottawa, ON K1Y 4E9 Canada; 2 Clinical Epidemiology Program (Centre for Transfusion Research), Ottawa Hospital Research Institute, Ottawa, ON Canada; 3 Department of Medicine, University of Montreal, Montreal, QC Canada; 4 Department of Anesthesiology & Critical Care, Université Laval, Quebec City, QC Canada; 5 Department of Medicine, Université Laval, Quebec City, QC Canada; 6 Deparment of Anesthesiology, Pharmacology & Therapeutics, University of British Columbia, Vancouver, BC Canada; 7 Department of Medicine, University of Manitoba, Winnipeg, MB Canada; 8 Department of Medicine, University of Ottawa, Ottawa, ON Canada; 9 Department of Medicine, University of Saskatchewan, Regina, SK Canada; 10 Department of Surgery (Neurosurgery), University of Ottawa, Ottawa, ON Canada; 11 Department of Social and Preventive Medicine, Université Laval, Quebec City, QC Canada; 12 Department of Surgery, University of Ottawa, Ottawa, ON Canada

**Keywords:** Subarachnoid hemorrhage, Cerebral aneurysm, Anemia, Red blood cell transfusion, Cohort study

## Abstract

**Background:**

Whether a restrictive strategy for red blood cell (RBC) transfusion is applied to patients with aneurysmal subarachnoid hemorrhage (aSAH) is unclear. To inform the design and conduct of a future clinical trial, we sought to describe transfusion practices, hemoglobin (Hb) triggers, and predictors of RBC transfusion in patients with aSAH.

**Methods:**

This is a retrospective cohort study of all consecutively admitted adult patients with aSAH at four tertiary care centers from January 1, 2012, to December 31, 2013. Patients were identified from hospital administrative discharge records and existing local aSAH databases. Data collection by trained abstractors included demographic data, aSAH characteristics, Hb and transfusion data, other major aSAH cointerventions, and outcomes using a pretested case report form with standardized procedures. Descriptive statistics were used to summarize data, and regression models were used to identify associations between anemia, transfusion, and other relevant predictors and outcome.

**Results:**

A total of 527 patients met inclusion eligibility. Mean (±SD) age was 57 ± 13 years, and 357 patients (67.7%) were female. The median modified Fisher grade was 4 (IQR 3–4). Mean nadir Hb was 98 ± 20 g/L and occurred on median admission day 4 (IQR 2–11). RBC transfusion occurred in 100 patients (19.0%). Transfusion rates varied across centers (12.1–27.4%, *p* = 0.02). Patients received a median of 1 RBC unit (IQR 1–2) per transfusion episode and a median total of 2 units (IQR 1–4). Median pretransfusion Hb for first transfusion was 79 g/L (IQR 74–93) and did not vary substantially across centers (78–82 g/L, *p* = 0.37). Of patients with nadir Hb < 80 g/L, 66.3% received a transfusion compared with 2.0% with Hb nadir ≥ 100 g/L (*p* < 0.0001). Predictors of transfusion were history of oral anticoagulant use, anterior circulation aneurysm, neurosurgical clipping, and lower Hb. Controlling for numerous potential confounders, transfusion was not independently associated with poor outcome.

**Conclusions:**

We observed that moderate anemia remains very common early in admission following SAH. Only one-fifth of patients with SAH received RBC transfusions, mostly in cases of significant anemia (Hb < 80 g/L), and this did not appear to be associated with outcome.

**Electronic supplementary material:**

The online version of this article (10.1186/s13054-018-2089-7) contains supplementary material, which is available to authorized users.

## Background

Most atraumatic subarachnoid hemorrhages are caused by a ruptured cerebral artery aneurysm (aneurysmal subarachnoid hemorrhage [aSAH]). Patients who survive the primary event are at high risk of complications, including delayed cerebral ischemia (DCI) such as with vasospasm, which may result in further neurologic deficits and increased likelihood of death [1]. Initial management focuses on patient stabilization and support; securing of the aneurysm; and monitoring for, preventing, and treating potential complications [2, 3]. Medical complications, including anemia, are common and affect up to 50% of patients with aSAH [4]. Among them, both anemia and transfusion of red blood cells (RBC) have been associated with complications and poor outcomes [4]. Anemic patients with aSAH are not only more likely to receive RBC transfusions but also at an increased risk of ischemic complications [4]. Those who receive RBC transfusions have been shown to have less favorable hospital outcomes, including severe disability [5].

Preclinical studies in brain injury suggest that RBC transfusion to treat anemia improves oxygen delivery [6]. Only one small trial (*N* = 44) has compared two transfusion targets (100 g/L and 115 g/L) in aSAH, but it was underpowered to examine clinically important outcomes [7]. Another small mixed brain injury population trial (*N* = 102) compared a hemoglobin (Hb) target with a transcranial oxygenation target, but it, too, was underpowered to examine clinically important outcomes [8]. Despite this absence of evidence, current aSAH management guidelines recommend considering RBC transfusion in anemic patients at risk for cerebral ischemia, but they do not suggest specific transfusion thresholds to guide clinicians [1, 3]. These recommendations are in contrast with current stated aSAH management practice derived from surveys [9] and evidence from clinical trials in other critically ill adult and pediatric populations, which support a more restrictive RBC transfusion approach [10, 11].

Canadian epidemiologic data on aSAH is sparse and more than 20 years old [12]. These data are limited to characterizing hospitalizations and case fatality rates with no published data on transfusion practices, which have changed significantly in critically ill patients over the last decade. In collaboration with the Canadian Critical Care Trials Group, we conducted a retrospective, multicenter cohort study of consecutive patients with aSAH to better understand current RBC transfusion practices.

## Methods

### Study design and objectives

We conducted a multicenter retrospective observational study of patients with aSAH admitted to one of four Canadian academic tertiary care hospitals in four different provinces. This study was approved by the local research ethics board of each participating center, which waived the need for informed consent.

Our a priori primary objective was to describe RBC transfusion practices, including the distribution of Hb values at which patients are transfused, the proportion of patients transfused, the median number of RBC transfusions per patient, the predictors of anemia and transfusion, and practice variations between participating centers. Our secondary objective was to examine the association of RBC transfusion and clinical outcome.

### Patient selection

We included all consecutive admissions for aSAH between January 1, 2012, and December 31, 2013. We identified potentially eligible patients by screening two sources: (1) hospital discharge abstracts, including all discharge abstracts that listed the diagnosis of primary SAH using International Classification of Diseases, Tenth Revision (ICD-10), codes (I60.0 to I60.9), and, where available, (2) local database or patient repositories, including all patients with a diagnosis of aSAH obtained from an existing local hospital, intensive care unit (ICU), or neurosurgical database. The local database had to include consecutive cases and be searchable by principal diagnoses that were not ICD code-generated.

Each patient underwent chart review by a trained data abstractor. For final inclusion in the cohort, patients had to be ≥ 18 years of age and to have sustained an aSAH. The diagnosis of aSAH required both evidence of SAH (by at least one of the following: blood in subarachnoid space as demonstrated by neuroimaging [e.g., computed tomography {CT}, magnetic resonance imaging, lumbar puncture demonstrating xanthochromia and/or > 5 × 10^6^ RBCs/L, or blood in subarachnoid space as observed at postmortem autopsy report) and (2) evidence of aneurysm rupture as the cause (by at least one of the following: aneurysm observed on angiography, aneurysm demonstrated on neuroimaging report [computed tomographic angiography or magnetic resonance angiography], or aneurysm observed on postmortem examination report). No exclusion criteria were used, because we wanted to describe all adult patients with true aSAH as established by the inclusion criteria. Similar criteria have been used in previous cohort studies examining anemia and RBC transfusion in aSAH [5, 13].

### Patient management

All four study centers are academic tertiary care referral hospitals with complete neurosurgical, neurointerventional, and intensive care services. Management of patients with aSAH was in accordance with local practice and procedures and the most recent aSAH management guidelines [3]. Specifically, acute care goals included early treatment of hydrocephalus and securing of ruptured aneurysm, as well as traditional management of intracranial hypertension with goal-directed osmotic therapy. All sites fostered early detection of DCI with close clinical observation with or without serial surveillance imaging (e.g., transcranial Doppler). Medical complications of aSAH, including cardiac dysfunction, electrolyte abnormalities, and temperature dysregulation, were managed according to local practice and in accordance with accepted standards.

At each site, acute care management of patients with aSAH occurred in either a level 3 ICU for mechanically ventilated and/or unstable patients or in a level 2 high-acuity unit until such time that the aneurysm was secured, the patient was off all life supports and vasoactive medications, and in the absence of DCI. During the study period, no participating center had a specific RBC transfusion protocol for managing aSAH, nor did any of them have specific transfusion guidelines, and the decision to initiate as well as the timing of RBC transfusion was at the discretion of the treating team.

### Data collection

Data collection was completed by trained data abstractors using a previously prepared and piloted case report form and a standardized operations manual tested for completeness, clarity, and ease of use. We used multiple sources, including electronic and paper medical records as well as laboratory and imaging reports. An ICU day was considered any amount of time in a single calendar day admitted to a level 2 or 3 unit (Additional file 1: Supplemental Material). SAH severity was captured with Hunt and Hess grade [14], World Federation of Neurological Surgeons grade [15], and/or the modified Fisher scale [16]. When not explicitly reported, modified Fisher scale grade was calculated using the first available CT scan.

The date and time of each RBC transfusion administered was recorded. Multiple units of RBCs transfused on the same calendar day were considered to be part of a single transfusion episode, unless separate transfusions occurred intraoperatively (this was considered a separate transfusion episode). A pretransfusion Hb (defined as the most recent Hb [within 48 h] drawn prior to the initiation of RBC transfusion) was captured for each transfusion episode. In the 6 h preceding an RBC transfusion, we identified episodes of active bleeding (defined as more than 250 ml of blood loss in 1 h or active blood loss associated with hemodynamic instability). We captured admission Hb, daily nadir Hb values for the first 21 days of admission, and hospitalization and ICU stay nadir Hb. We defined anemia as a Hb value less than or equal to 100 g/L because this value has clinical significance and represents an important threshold in multiple previously published transfusion trials [11, 17–19]. Diagnostic criteria for vasospasm and cerebral infarction were established a priori (Additional file 1: Supplemental Material).

We recorded vital status at discharge, discharge destination (home, other hospital, rehabilitation, long-term care/nursing home, or other), and functional status at discharge applying the modified Rankin Scale (mRS) criteria (from 0 to 6) (Additional file 1: Supplemental Material). Functional status was ascertained using available documentation, including physiotherapy and occupational therapy discharge notes and rehabilitation assessments. We dichotomized status at discharge to good (mRS 0–3) or poor (mRS 4–6) outcome.

### Statistical analyses

Descriptive statistics were used to summarize the study data. Continuous variables are presented as mean with SD or median with IQR, depending on their distribution. Frequency and proportion estimates are presented as point estimates with 95% CIs. We used the chi-square or Fisher’s exact test, as appropriate, to examine differences in transfusion rates among different trigger thresholds. Predictors of anemia, RBC transfusion, and poor neurologic functional outcome were tested using a random effects generalized linear model to account for clustering at the center level. Potential predictor variables for each model were set a priori and included variables identified previously in the literature and those with clinical significance. For anemia, these included admission Hb, age, gender, history of oral anticoagulant, modified Fisher grade, aneurysm size, method of securing the aneurysm (clip vs coil), and presence of vasospasm (prior to onset of anemia). For RBC transfusion, predictors considered included age, gender, history of oral anticoagulant, modified Fisher grade, location of aneurysm (anterior vs posterior circulation), method of securing the aneurysm (clip vs coil), admission Hb, presence of anemia, and vasospasm or cerebral infarction (prior to the first RBC transfusion). Owing to the clinical correlation between the latter two, only vasospasm was considered in the final model. Finally, predictors of poor outcome entered in the model included all of the previously mentioned variables and other preexisting comorbidities, presenting Glasgow Coma Scale (GCS) score, need for mechanical ventilation, hospital length of stay, need for ICU admission, need for tracheostomy, or need for percutaneous gastrostomy tube. Similarly, given the clinical correlation between the need for mechanical ventilation, need for ICU admission, and need for tracheostomy, we did not include need for ICU admission in the final model, because it was least significant in univariate analysis. Cerebral infarction was included (instead of vasospasm) owing to its significant correlation during univariate analysis. We examined for multicollinearity among predictors. Statistical interaction between certain variables was considered and included in the model only if statistically significant. Statistical significance was considered as a *p* value < 0.05. All analyses were completed using SAS 9.3 software (SAS Institute, Cary, NC, USA).

## Results

Of the 886 screened patients, 42 had no SAH, and 317 had SAH resulting from causes other than aneurysm rupture, leaving a cohort of 527 patients. Baseline and disease-specific characteristics, cointerventions, and disease-related complications are presented in Table 1.

**Table 1:** Patient characteristics, interventions, and complications

	Data (*N* = 527)
Characteristics	
Age, yr, mean ± SD	57 ± 13
Female, no. (%)	357 (67.7)
Comorbidities, no. (%)	
Hypertension	233 (44.2)
Heart disease	41 (7.8)
Active tobacco smoker	185 (35.1)
Home medications, no. (%)	
Antiplatelet	67 (12.7)
Oral anticoagulant	20 (3.8)
Statin	100 (19.0)
aSAH characteristics	
Presenting GCS, mean ± SD	11 ± 5
Modified Fisher scale score, median (IQR)	4 (3–4)
Aneurysm	
Size, mm, mean ± SD	6.9 ± 4.3
Posterior circulation, no. (%)	182 (34.5)
Anterior circulation, no. (%)	327 (62)
Interventions and cointerventions, no. (%)	
Surgical clipping	209 (39.7)
Endovascular coiling	275 (52.5)
Postadmission day aneurysm secured, median (IQR)^a^	0 (0–1)
EVD	236 (44.8)
RBC transfusion	100 (19.0)
Need for mechanical ventilation	216 (49.8)
Complications, no. (%)	
Vasospasm	142 (26.9)
New ischemic neurologic lesions	104 (19.7)

*Abbreviations: aSAH* Aneurysmal subarachnoid hemorrhage, *EVD* Externalized ventricular drain, *GCS* Glasgow Coma Scale, *RBC* Red blood cells

^a^Eleven dates missing

### Anemia

Anemia at presentation was found in 29 patients (5.5%). Among the other 498 patients, the hospitalization incidence of acquired anemia was 52.0% (47.6–56.4%). The overall prevalence of anemia within the first 21 days of admission was 51.8% (95% CI 47.5–56.1%). The mean hospitalization nadir Hb was 98.0 ± 19.5 g/L and occurred on median admission day 4 (IQR 2–11). The proportion of anemic patients varied across centers (*p* = 0.005) (Additional file 1: Figure S1). Female sex, history of oral anticoagulant use, modified Fisher grade 3 or 4, admission Hb, and aneurysm secured by neurosurgical clipping were independent predictors of anemia (*see* Table 2).

**Table 2:** Predictors of anemia (Hb ≤ 100 g/L) during admission for subarachnoid hemorrhage, clustered by center

Univariate analysis				Multivariable model (*n* = 437; 238 events)			
Variable	OR	95% CI	*p* Value	Variable	OR	95% CI	*p* Value
Age (increase by 10 yr)	1.02	0.97–1.08	0.472	Age (increase by 10 years)	0.99	0.91–1.09	0.913
Sex (female vs other)	2.82	1.76–4.50	< 0.0001	Sex (female vs other)	1.91	1.08–3.40	0.027
Hx of oral AC use	6.17	2.79–14.97	< 0.0001	Hx of oral AC use	5.98	2.11–16.95	0.001
Admission hemoglobin (increase by 10 g/L)	0.35	0.32–0.39	< 0.0001	Admission hemoglobin (increase by 10 g/L)	0.55	0.43–0.71	< 0.0001
Modified Fisher grade 3–4 vs 0–2^a^	1.42	0.98–2.08	0.067	Modified Fisher grade 3–4 vs 0–2^a^	1.96	1.07–3.57	0.028
Anterior circulation (vs posterior)	0.98	0.65–1.48	0.929	Anterior circulation (vs posterior)	0.58	0.30–1.13	0.107
Aneurysm size (increase by 1 mm)	1.03	1.02–1.05	< 0.0001	Aneurysm size (increase by 1 mm)	1.03	0.98–1.07	0.226
Clip (vs other)	3.19	2.09–4.85	< 0.0001	Clip (vs other)	4.62	2.46–8.69	< 0.0001
Vasospasm (preanemia)	0.78	0.52–1.17	0.223	Vasospasm (preanemia)	1.49	0.79–2.80	0.214

*Abbreviations: AC* Anticoagulant, *antiplt* Antiplatelet, *Hb* Hemoglobin, *Hx* History, *RBCTx* Red blood cell transfusion, *SAH* Subarachnoid hemorrhage

^a^Dichotomized as high (3–4) vs low (0–2) grade

### Primary outcome: RBC transfusion practices

Overall, 100 patients (19.0%, 95% CI 15.6–22.3%) underwent transfusion of at least 1 unit of packed red blood cells (PRBCs). A median of 1 unit of PRBCs (IQR 1–2) per transfusion episode was received, and a median of 2 units of PRBCs (IQR 1–3) was received during the hospitalization. The median pretransfusion Hb of the first transfusion was 79 g/L (IQR 74–93), and the median was 80 g/L (IQR 72–86) for all transfusions. A total of 35 patients (6.6%) had an intraoperative transfusion, and 24 patients (4.6%) received ≥1 unit of PRBCs within 6 h of active bleeding.

**Fig. 1:**
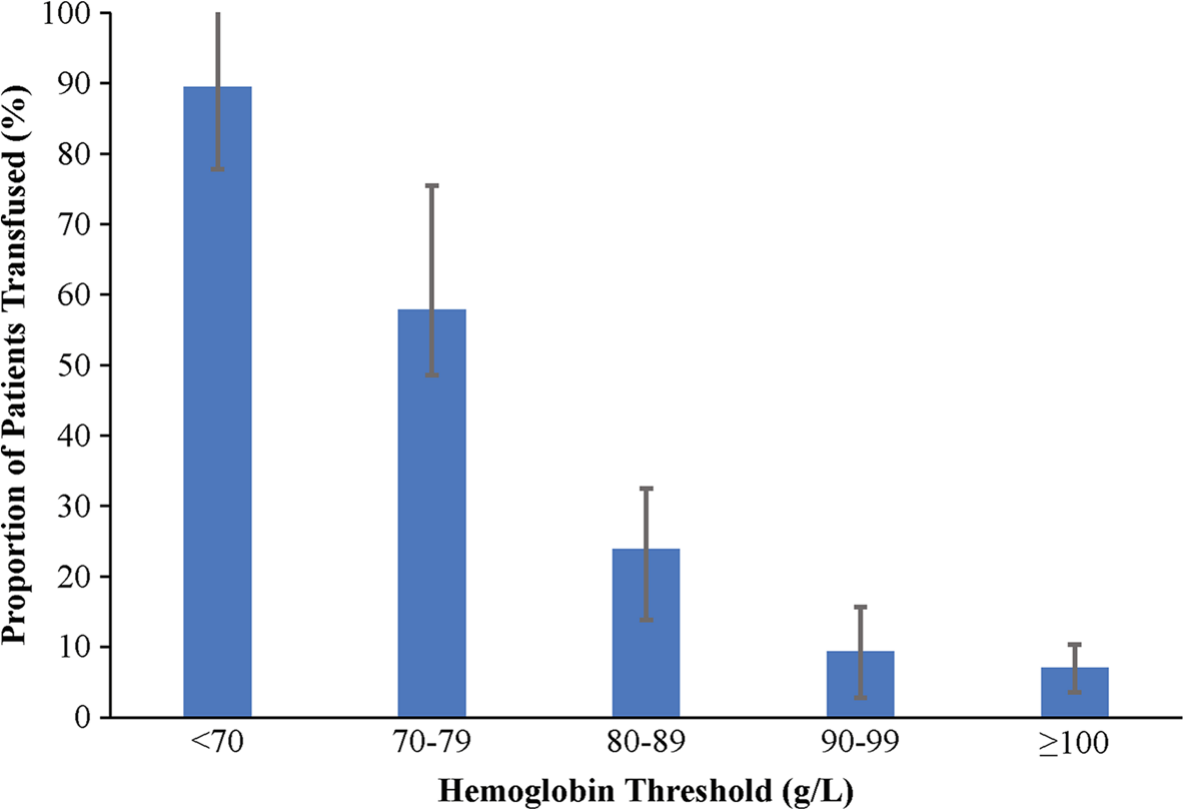
Red blood cell transfusion rates across hemoglobin thresholds. Proportion of patients transfused according to hemoglobin threshold. Error bar depicts proportion 95% CI

We grouped patients by their Hb nadir (in nontransfused patients) and pretransfusion Hb of first transfusion (in transfused patients) in Hb increments of 10 g/L. RBC transfusion rates were highest in those patients with a Hb < 80 g/L (*p* < 0.0001) (Fig. 1). Among patients with a nadir Hb of 100 g/L or higher, only 7.0% of patients received an RBC transfusion.

**Fig. 2:**
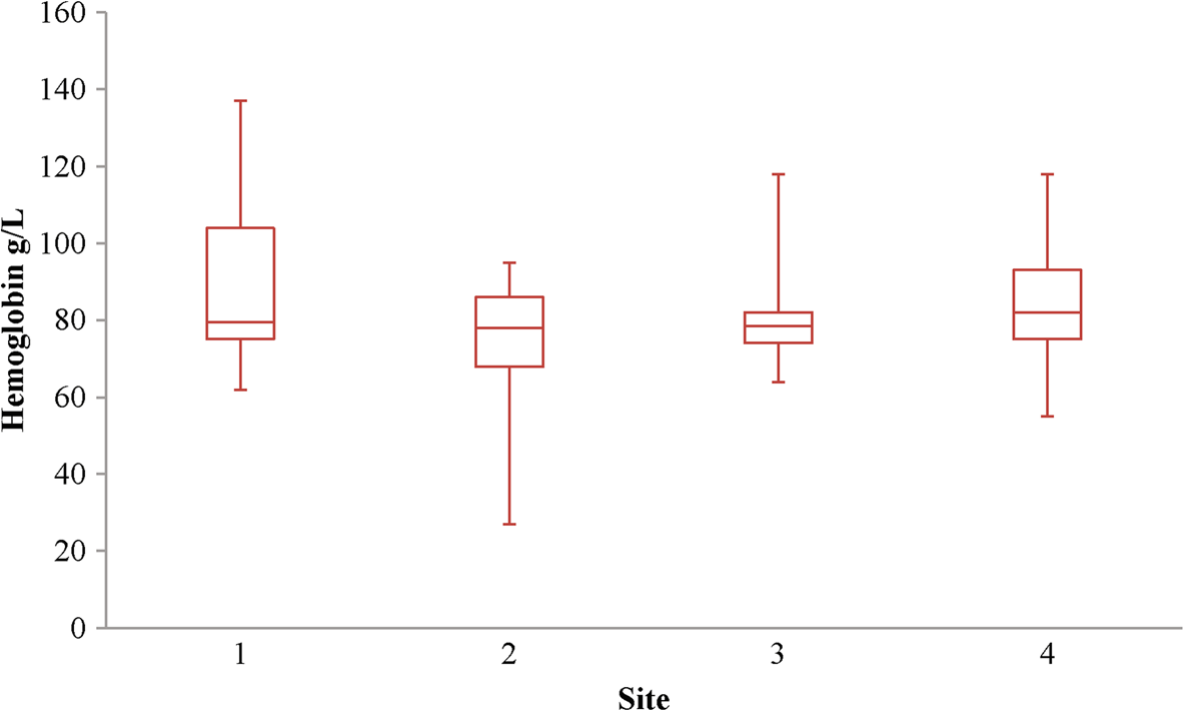
Red blood cell transfusion threshold site variation. Whisker box plot of pretransfusion hemoglobin according to site. The center line in the box represents the median, and the outside lines of the box represent the first and third quartiles. The lines extending from the boxes demonstrate the minimum and maximum values

Transfusion rates varied from 12.5% to 27.4% across participating sites (*p* = 0.016) (Additional file 1: Figure S2). However, median pretransfusion Hb did not vary significantly between sites, ranging from 78 to 82 g/L (*p* = 0.37) (Fig. 2).

### Predictors of RBC transfusion

Univariate predictors of at least 1 RBC transfusion included history of home anticoagulant use, lower admission Hb, ruptured aneurysm in the anterior cerebral circulation, secured aneurysm by neurosurgical clipping, and anemia (Table 3). In our regression model, only lower admission Hb, ruptured aneurysm secured by neurosurgical clipping, and anemia remained independent risk factors for transfusion. Among patients with vasospasm, the only predictor of transfusion was moderate anemia (OR 9.59, 95% CI 2.75–33.48, *p* = 0.0004).

**Table 3:** Predictors of one or more red blood cell transfusions during admission for subarachnoid hemorrhage, clustered by center

Univariate analysis				Multivariable model (*n* = 463; 95 events)			
Variable	OR	95% CI	*p* Value	Variable	OR	95% CI	*p* Value
Age (increase by 10 years)	1.07	0.90–1.28	0.458	Age (increase by 10 years)	1.06	0.78–1.45	0.711
Sex (male vs other)	0.45	0.19–1.10	0.080	Sex (male vs other)	0.84	0.45–1.57	0.593
Hx of oral AC use	1.93	1.01–3.68	0.046	Hx of Oral AC use	1.00	0.60–1.67	0.993
Admission Hb (increase by 10 g/L)	0.40	0.39–0.41	< 0.0001	Admission Hb (increase by 10 g/L)	0.81	0.69–0.96	0.013
Modified Fisher grade 3–4 vs 0–2^a^	1.02	0.90–1.15	0.765	Modified Fisher Grade 3–4 vs 0–2^a^	1.02	0.77–1.36	0.876
Anterior circulation (vs posterior)	1.46	1.17–1.83	0.001	Anterior circulation (vs Posterior)	1.18	0.90–1.55	0.231
Clip (vs other)	4.39	3.25–5.92	< 0.0001	Clip (vs other)	2.44	1.21–4.94	0.013
Anemia (Hb ≤100 g/L)	28.17	11.06–71.75	< 0.0001	Anemia (Hb ≤100 g/L)	17.38	5.11–59.13	< 0.0001
Vasospasm (pre-RBCTx)	1.62	0.91–2.88	0.100	Vasospasm (pre-RBCTx)	1.11	0.40–3.09	0.845
Cerebral infarct (pre-RBCTx)	1.08	0.96–1.23	0.206				

*Abbreviations: AC* Anticoagulant, *antiplt* Antiplatelet, *Hb* Hemoglobin, *Hx* History, *RBCTx* Red blood cell transfusion

^a^Dichotomized as high (3–4) vs low (0–2) grade

We stratified patients on the basis of their nadir Hb to examine differences in transfusion predictors. Among the most severely anemic patients (Hb < 80 g/L, *n* = 98), 66.3% (65 patients; 95% CI 57.0–75.7%) of patients received a transfusion with no identifiable significant predictors in our model (Table 4). In the moderate anemia group (80 ≤ Hb < 100 g/L, *n* = 175), 17.1% (30 patients; 95% CI 11.6–22.7%) received a transfusion. Independent predictors of transfusion in this group were increasing age (per 10-year increase OR 1.18, 95% CI 1.10–1.26), male sex (OR 1.42, 95% CI 1.03–1.96), and neurosurgical clipping of the aneurysm (OR 4.60, 95% CI 1.89–11.17). Of the 254 patients in the nonanemic group (nadir Hb ≥ 100 g/L), 5 patients (2.0%, 95% CI 0.6–4.5%) received a transfusion. There were insufficient events to identify independent predictors of transfusion in this group.

**Table 4:** Predictors (multivariable model) of red blood cell transfusion, stratified by nadir hemoglobin

Variable	Hb < 80 g/L (*n* = 83; 61 RBCTx events)			80 ≤ Hb < 100 g/L (*n* = 168; 32 RBCTx events)		
	OR	95% CI	*p* Value	OR	95% CI	*p* Value
Age (increase by 10 years)	1.03	0.82–1.29	0.801	1.18	1.10–1.26	< 0.0001
Male sex (vs other)	0.48	0.13–1.87	0.293	1.42	1.03–1.96	0.031
Fisher grade 3–4 (vs 0–2)	1.29	0.76–2.18	0.347	0.64	0.29–1.42	0.268
Anterior circulation (vs posterior)	1.77	0.65–4.79	0.262	0.85	0.60–1.22	0.385
Clip (vs other)	1.00	0.16–6.18	1.000	4.60	1.89–11.17	0.001
Vasospasm (pre-RBCTx)	0.81	0.23–2.91	0.751	1.57	0.68–3.62	0.294

*Hb* Hemoglobin, *RBCTx* Red blood cell transfusion

### RBC transfusion association with outcome

Neurologic functional outcome at discharge was missing for 66 patients (12.5%). By hospital discharge, 93 (20.2%) patients had died and 220 (47.7%) had a poor neurologic outcome (mRS 4–6). Although both anemia (Hb < 100 g/L) and RBC transfusion were predictors of poor outcome (ORs 2.44 and 3.72, 95% CIs 1.67–3.57 and 2.23–6.19, respectively), neither of them was a significant independent predictor when controlling for these and other factors that are likely to affect outcome (Table 5). Independent predictors of poor neurologic outcome included increasing age; history of hypertension, stroke, or ever smoking; home oral anticoagulant use, high-grade modified Fisher grade; anterior circulation aneurysm; poor GCS at presentation; need for mechanical ventilation; and need for percutaneous gastrostomy tube (Table 4).

**Table 5:** Predictors of poor outcome^a^, clustered by center

Univariate analysis				Multivariable model (*n* = 404; 199 events)			
Variable	OR	95% CI	*p* Value	Variable	OR	95% CI	*p* Value
Age (increase by 10 years)	1.65	1.28–2.12	0.0001	Age (increase by 10 year)	1.58	1.30–1.93	< 0.0001
Sex (male vs other)	0.92	0.71–1.20	0.548	Sex (male vs other)	0.96	0.35–2.63	0.936
History of hypertension	2.06	1.56–2.72	< 0.0001	History of Hypertension	1.47	1.06–2.03	0.022
History of ischemic stroke	9.22	3.81–22.35	< 0.0001	History of Ischemic stroke	7.85	2.29–26.92	0.001
History of heart disease	2.99	1.40–6.41	0.005	History of Heart disease	1.02	0.33–3.12	0.976
Smoking history	0.54	0.38–0.78	0.0009	Smoking history	0.58	0.34–0.99	0.047
Home oral anticoagulant	8.44	1.22–58.54	0.031	Home Oral anticoagulant	5.38	1.48–19.64	0.011
Fisher grade 3–4 vs 0–2	2.83	2.31–3.45	< 0.0001	Fisher Grade 3–4 vs 0–2	1.60	1.45–1.77	< 0.0001
Anterior circulation (vs posterior)	1.17	1.00–1.37	0.056	Anterior circulation (vs Posterior)	1.54	1.10–2.14	0.011
GCS (for each increase by 1)	0.82	0.81–0.84	< 0.0001	GCS (for each increase by 1)	0.95	0.90–1.00	0.045
Need for mechanical ventilation	9.58	6.89–13.31	< 0.0001	Need for Mechanical Ventilation	4.94	3.14–7.78	< 0.0001
Clip (vs other)	0.86	0.48–1.54	0.602	Clip (vs other)	0.58	0.24–1.42	0.233
RBCTx	3.72	2.23–6.19	< 0.0001	RBCTx	1.50	0.83–2.71	0.175
Anemia (Hb ≤ 100 g/L)	2.44	1.67–3.57	< 0.0001	Anemia (Hb ≤100 g/L)	1.06	0.57–1.97	0.860
Vasospasm	1.35	0.84–2.18	0.217	Cerebral Infarct	1.78	0.75–4.21	0.188
Cerebral infarct	2.83	1.73–4.65	< 0.0001	Hospital length of stay	1.00	0.99–1.00	0.456
Hospital length of stay	1.01	1.00–1.02	0.016	Need for tracheostomy	1.97	0.45–8.57	0.365
Need for ICU/IMCU admission	1.38	0.48–3.92	0.549	Need for PEG tube	8.62	1.12–66.56	0.039
Need for tracheostomy	7.96	2.89–21.89	< 0.0001				
Need for PEG tube	25.22	6.28–101.37	< 0.0001				

*Abbreviations: GCS* Glasgow Coma Scale, *Hb* Hemoglobin, *ICU* Intensive care unit, *IMCU* Intermediate care unit, *PEG* Percutaneous gastrostomy tube, *RBCTx* Red blood cell transfusion

^a^67 missing

## Discussion

In this multicenter retrospective review of patients with aSAH, we found that although moderate anemia incidence remains very high (51.8%), RBC transfusion is uncommon (19.0%). Significant RBC transfusion rates were only observed with severe anemia (Hb ≤ 80 g/L), suggesting a “restrictive” RBC transfusion practice among the hospitals in the study. Although the proportion of patients transfused differed across participating sites, the median pretransfusion Hb did not differ, ranging from 78 to 82 g/L. In this large and robust dataset, controlling for other clinical factors that may influence outcome, RBC transfusion was not correlated with negative outcomes. Further, although our data are not able to demonstrate if RBC transfusion is in fact helpful in the improvement of neurologic outcome, in the current cohort, it is a marker of disease severity.

Our findings are important and add significantly to the aSAH and transfusion literature for several reasons. First, this is the first multi-institutional study examining RBC transfusion practice in this patient population. Second, we have described independent predictors of RBC transfusion using a robust dataset. Further, we have attempted to describe how these factors may change depending on the level of Hb of a given patient. Last, we have demonstrated with a large, multicenter dataset populated with a large number of variables that, when controlling for other important factors, RBC transfusion does not appear to be a predictor of poor neurologic functional outcome at hospital discharge, but we cannot conclude that it is either harmful or helpful in this population.

The common occurrence of anemia following aSAH has been previously described [4, 5]. Despite changes in common management strategies of patients with aSAH since these earlier descriptions, including a movement away from hypervolemia and hemodilution (two of the so-called triple-H therapies) in favor of hyperdynamic therapy for DCI management [20], our findings suggest that this has had little impact on anemia incidence. Nonetheless, our findings also suggest that RBC transfusion is uncommon relative to the incidence of anemia and is largely restricted to those with severe anemia (median Hb ≤ 80 g/L). This “restrictive RBC transfusion” practice is in keeping with a stated practice from a 2010 North American survey [21]. Our observed practice is vastly different from the two transfusion triggers (100 g/L vs 115 g/L) employed in the only randomized controlled trial examining RBC transfusion thresholds in patients with aSAH and from the current aSAH management guidelines, which include a recommendation to consider RBC transfusion in anemic patients at risk for cerebral ischemia [3].

Four prior studies [5, 22–24] and an abstract publication [25] report adjusted analyses of the effect of RBC transfusion on poor outcome, using a variety of control variables. Additionally, three prior studies [5, 26, 27] (and two potentially related abstracts [28, 29]) report adjusted analyses of effect of RBC transfusion on mortality. Two demonstrated a statistically significant association:

1. RBC transfusion increased the odds of death by threefold (OR 3.16, 95% CI 1.02–9.69) when controlling for nadir Hb (and its interaction with RBC transfusion) in an RBC transfusion propensity score analysis of predicted mortality using a cohort from two hospitals [26]

2. A fourfold increase in odds of death or poor neurologic outcome was seen with RBC transfusion (OR 4.3, 95% CI 1.9–9.3) in a single-center cohort when controlling for age, SAH severity, and anemia [5].

The differences found in our study may be attributable to its larger, more current sample, higher event rates, and multicenter contribution to the cohort, distinguishing it from all of these previous studies.

The strengths of this study lie in the rigorous methodology employed to minimize some of the biases inherent in retrospective cohort studies. Specifically, we set the protocol a priori and undertook data collection by trained abstractors using a piloted data collection tool and a set of procedures and definitions. We made a significant effort to ensure complete data abstraction with multiple data sources. These efforts led to our ability to gather robust data that allowed for controlled analyses that have limited other studies.

Our study is not without limitation, however. We cannot eliminate all of the inherent potential biases. Although we were successful in obtaining modified Fisher grades for the majority of patients, the documenting of other prognostic scales was poor, limiting our ability to use these data in our models. We applied very stringent and conservative diagnostic criteria for vasospasm, as a measure of DCI, that included the need for imaging, clinical change, and treatment initiation. Owing to variances and inadequacies in the documentation, we believe that our finding of a vasospasm incidence of 26.9% is a significant underestimation of the true incidence. As such, this may have precluded our ability to demonstrate vasospasm as an important predictor of RBC transfusion, both overall and in the anemia subgroups. Further, we were unable to collect undocumented factors that may have led to the decision to transfuse a patient to the extent that it is unclear if or to what degree a single Hb value influenced the decision. Last, as we studied a retrospective cohort, we were limited to measuring outcome at hospital discharge because 6- or 12-month follow-up was not ascertained. It is clear that recovery from aSAH occurs over months and even years, and this shorter follow-up may bias toward worse outcome [30, 31].

## Conclusions

Our study suggests that current practice includes a restrictive transfusion strategy and that RBC transfusion in this cohort of patients was not associated with a worse outcome when controlling for other important factors. Rigorous randomized controlled trials to better understand the role and timing of RBC transfusion in this patient population are still needed.

## Additional file

Additional file 1: Supplemental methods: expanded definitions. Supplemental data: oral anticoagulant data. **Figure S1.** Anemia (hemoglobin, ≤100 g/L) rates across centers. **Figure S2.** RBC transfusion rates across centers. (DOCX 70 kb)

## Abbreviations

aSAH: Aneurysmal subarachnoid hemorrhage
CT: Computed tomography
DCI: Delayed cerebral ischemia
GCS: Glasgow Coma Scale
Hb: Hemoglobin
ICU: Intensive care unit
mRS: Modified Rankin Scale
PEG: Percutaneous gastrostomy tube
PRBCs: Packed red blood cells
RBC: Red blood cell
