# Supplementary material for: Anemia prevalence and incidence and red blood cell transfusion practices in aneurysmal subarachnoid hemorrhage: results of a multicenter cohort study
Source: Crit Care. 2018 Jul 4;22:169. doi: 10.1186/s13054-018-2089-7 (PMC6031110; doi:10.1186/s13054-018-2089-7)
Supplement: Supplementary file 1 — Supplemental methods: expanded definitions. Supplemental data: oral anticoagulant data. Figure S1. Anemia (hemoglobin, ≤100 g/L) rates across centers. Figure S2. RBC transfusion rates across centers. (DOCX 70 kb) [file 13054_2018_2089_MOESM1_ESM.docx]

**Supplemental Material**

**Contents:**

Supplemental Methods: Expanded Definitions

Supplemental Data: Oral Anticoagulant Data

Supplemental Figure I: Anemia (Hemoglobin ≤100g/L) rates across centres

Supplemental Figure II: RBC transfusion rates across centres

**Supplemental Methods: Expanded Definitions**

**Cerebral infarction:** a new hypodensity on CT imaging, perfusion abnormality on CT perfusion consistent with infarct or diffusion abnormality on MRI that occurred ≥ 4days after original SAH, and was not thought to be directly related to a clip or coil procedure.

**ICU day:** attributed to any amount of time in a single calendar day admitted to either a level 2 or 3 unit (i.e. any unit capable of administering life supporting therapies including vasoactive medications and/or ventilatory support)

**Modified Rankin Scale Scores:**

1. No symptoms
2. No disability despite symptoms, eg: able to carry out all usual duties and activities
3. Slight disability, eg: unable to do previous activities, but able to look after own affairs without assistance
4. Moderate disability, eg: requiring some help, but able to walk without assistance
5. Moderately severe disability, eg: unable to walk and to attend own needs without assistance
6. Severe disability, eg: bedridden, incontinent and requiring constant nursing care and attention
7. Dead

**Vasospasm:** the presence of all three of: a) a change in neurologic status not attributable to another etiology (eg: hydrocephalus, seizure); b) having vascular imaging (CT, MR, angio) performed after onset of symptoms and interpreted as demonstrating vasospasm; and c) having symptoms sufficiently severe and persistent to initiate treatment. Cerebral infarction was

**Supplemental Data: Oral Anticoagulant Data**

There were 20 patients in the cohort identified as having been on oral anticoagulants. Six of these individuals received an RBC transfusion (30%) and 16 (89%) overall had a poor neurological outcome (5 of whom had had a transfusion).

Supplemental Figure I. Anemia (hemoglobin ≤100g/L) rates across centres

Proportion of anemic patients across participating sites. Error bar depicts proportion 95% confidence interval.

Supplemental Figure II. RBC transfusion rates across centres

Proportion of patients transfused across participating sites. Error bar depicts proportion 95% confidence interval.
